# Detecting host responses to microbial stimulation using primary epithelial organoids

**DOI:** 10.1080/19490976.2023.2281012

**Published:** 2023-11-22

**Authors:** Jette Bornholdt, Christina V. Müller, Maria Juul Nielsen, Jesper Strickertsson, Daria Rago, Yun Chen, Grzegorz Maciag, Jonathan Skov, Anja Wellejus, Pawel J. Schweiger, Stine L. Hansen, Christa Broholm, Ismail Gögenur, Martti Maimets, Stine Sloth, Jakob Hendel, Adam Baker, Albin Sandelin, Kim B. Jensen

**Affiliations:** a Biotech Research and Innovation Centre, University of Copenhagen, Copenhagen, Denmark; b Department of Biology, University of Copenhagen, Copenhagen, Denmark; c Human Health Research, Chr. Hansen AS, Hørsholm, Denmark; d Novo Nordisk Foundation Center for Stem Cell Medicine, reNEW, University of Copenhagen, Copenhagen, Denmark; e Center for Surgical Science, Department of Surgery, Zealand University Hospital, Koge, Denmark; f Department of Clinical Medicine, University of Copenhagen, Copenhagen, Denmark; g Department of Gastroenterology, Herlev Hospital, University of Copenhagen, Copenhagen, Denmark

**Keywords:** Intestinal organoids, probiotics, microbiome, intestinal epithelium, bacterial–epithelial interactions

## Abstract

The intestinal epithelium is constantly exposed to microbes residing in the lumen. Traditionally, the response to microbial interactions has been studied in cell lines derived from cancerous tissues, e.g. Caco-2. It is, however, unclear how the responses in these cancer cell lines reflect the responses of a normal epithelium and whether there might be microbial strain-specific effects. To address these questions, we derived organoids from the small intestine from a cohort of healthy individuals. Culturing intestinal epithelium on a flat laminin matrix induced their differentiation, facilitating analysis of microbial responses via the apical membrane normally exposed to the luminal content. Here, it was evident that the healthy epithelium across multiple individuals (*n* = 9) demonstrates robust acute both common and strain-specific responses to a range of probiotic bacterial strains (BB-12^Ⓡ^, LGG^Ⓡ^, DSM33361, and Bif195). Importantly, parallel experiments using the Caco-2 cell line provide no acute response. Collectively, we demonstrate that primary epithelial cells maintained as organoids represent a valuable resource for assessing interactions between the epithelium and luminal microbes across individuals, and that these models are likely to contribute to a better understanding of host microbe interactions.

## Introduction

The gastrointestinal tract is responsible for digestion and absorption of nutrients from our diet. This process is supported by trillions of microbes that naturally inhabit the intestinal lumen. The small intestine and colon are composed of different cell types supporting the transfer of nutrients to the rest of the body through a single-layered epithelium that face the luminal content and secrete enzymes involved with digestion and absorption of nutrients. The epithelial layer is constantly in proximity to luminal microbes, and interactions with the intestinal epithelial cells represent a key element for sustained maintenance of the small intestine and the colon.

Numerous studies have outlined the crosstalk and interactions between the intestinal epithelium and commensal microbes.^1,2^ Animal studies have demonstrated that metabolites from the microbial digestion of nutrients influence the epithelial cells including short-chain fatty acids promoting differentiation of colonic epithelial cells,^3^ microbial-derived polyamines promoting proliferation,^4^ indole-3-lactic acid dampening inflammation in both epithelial cells and macrophages,^5^ inositol-1,4,5-trisphosphate supporting epithelial cell division^6^ and cell wall components stimulating general growth.^7^ Thus, there appears to be a complex interplay between microbes and the epithelium controlling its turnover, maintenance, and resilience against challenges.

It has been exceedingly difficult to study how non-transformed primary human epithelial cells interact with microbes, and most of our understanding stems from studies of colorectal cancer cell lines, in particular Caco-2 cells. This cell line forms a confluent and impermeable epithelial layer, when cultured on plastic or in a transwell system, and has been used extensively as a model of the intestinal epithelium, showing evidence of multilineage differentiation. However, given that Caco-2 is an immortalized cancer cell line with mutations in key regulatory genes, it is unclear how well Caco-2 cells mimic a normal epithelium.^8^

Primary intestinal epithelial cells isolated from biopsy material can be cultured as organoids under defined cell culture conditions in matrigel.^9^ Organoids grow as a polarized single-layered epithelium, with the basolateral surface facing the outside of the organoid, whereas the luminal apical surface faces the inside of the organoids.^10^ Interactions between microbes and primary intestinal epithelial cells have been studied by injection of microbes into the lumen of the organoids.^11–13^ Recent reports have explored the ability to grow intestinal epithelial cells as 2D cultures in extracellular matrix coated plastic surfaces and on raised inserts.^2,14^ These methods provide exquisite opportunities for monitoring cellular heterogeneity and cell signaling across large surfaces.^15–17^ This opens opportunities for studying interactions with the apical cell surface as this is facing toward the culture medium.^18^

Aside from the choice of cell models, an important but largely unstudied aspect is how varied microbe exposure response in primary intestinal epithelial cells is between individuals. This is relevant both for pathogenic bacteria and bacteria used for probiotic supplements. Interestingly, in a clinical trial, we previously demonstrated pronounced differences in the acute response to the *Lacticaseibacillus rhamnosus* LGG^Ⓡ^ (DSM33156 – formerly known as *Lactobacillus rhamnosus* LGG^Ⓡ^, hereafter called LGG^Ⓡ^) probiotic in healthy individuals, most likely driven by a difference in B-cell responsiveness,^19^ based on RNA sequencing of gut wall biopsies. While valuable, gut biopsies are not tractable for either mechanistic investigations or larger-scale screening. This motivates targeted studies of microbiotic responses across multiple individuals *in vitro*, using state-of-the-art organoid cell models.

Here, we set out to develop an *in vitro* cell culture platform that would enable comprehensive studies of interactions between epithelial cells and microbes. This would allow us to address whether there are differences in the acute epithelial response upon exposure to microbes between individuals. We generated a selection of organoid lines from the small intestine of 27 young healthy individuals. We selected optimized growth conditions for the cultures and established a 2D culture system, which over a defined period allowed us to establish confluent layers of primary epithelial cells. Interestingly, coculturing with a selection of different probiotic bacterial strains developed for food supplements promotes robust common and strain-specific transcriptional effects beyond what can be observed in established cancer cell lines.

## Results

### Establishment of organoid lines from healthy individuals

**Figure 1.**
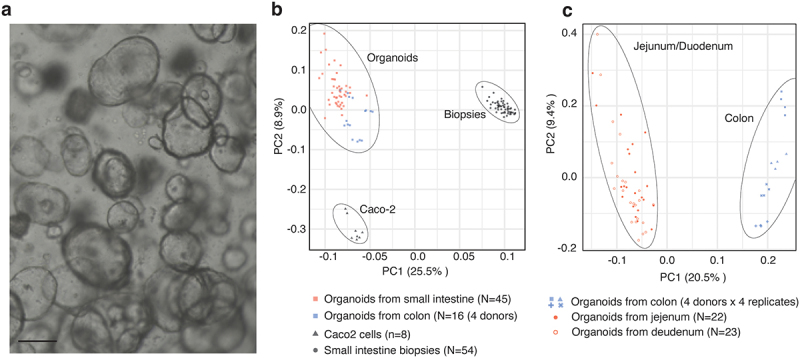
Derivation of organoid lines from a cohort of healthy individuals.

A cohort of 27 healthy individuals underwent endoscopy of the duodenum and proximal jejunum of the small intestine and biopsies were isolated from both sites, as described previously.^19^ Epithelial cells were subsequently extracted from these biopsies to generate an organoid bank representing the duodenum and jejunum from 23 and 22 healthy individuals, respectively (Figure 1a).^9^ In addition to the organoid lines derived from the small intestine, four organoid lines were similarly derived from the colon from an independent healthy cohort. RNA-seq expression analysis of the colonic and small-intestinal-derived organoids and corresponding biopsies from the small intestine and Caco-2 cells revealed substantial differences and clear separations between the three sample types when visualized using principal component analysis (PCA) (Figure 1b). While the cluster of biopsy samples was the most dissimilar to the others (likely reflecting their complex cellular composition), Caco-2 and organoid samples were also highly separated. Although all organoid samples clustered together, they had a higher dispersion. A subsequent PCA, only analyzing organoid samples, showed a clear separation between organoids derived from the duodenum and jejunum versus the colon (Figure 1c), while jejunum and duodenum organoids did not form distinct PCA clusters. Although, previous reports have described transcriptional differences between cultured epithelial cells from the different parts of the intestinal tract,^20,21^ there were no major expression differences between organoids derived from the duodenum and the proximal part of jejunum. In contrast, there were clear differences between organoids derived from the small and large intestines.

(a) Organoids derived from small intestinal biopsies (scale bar: 100 µm). (b) Principal component analysis (PCA) of data from RNAseq analysis of organoids (squares) derived from the small intestine (red squares *N* = 45) and colon (blue squares; *N* = 4 donors in quadruplicate), small intestinal biopsies (circles; *N* = 54), and the Caco-2 cell line (triangles *N* = 8). PC 1 and 2 are shown on X and Y axes, and percent variance is indicated. (c) PCA of data from RNAseq analysis of organoids derived from the small intestine (red filled circles: jejunum, *N* = 22; red stroke circles: duodenum, *N* = 23) and the colon only (blue *N* = 4 donors in quadruplicate: donors are indicated by shape). X and Y axes show PC 1 and 2, and percent variance is indicated.

### Transfer of organoid lines to Intesticult™

**Figure 2.**
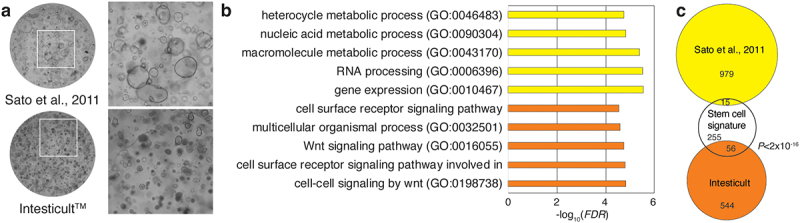
Characterization of impact of media composition on organoid cultures.

Several different cell culture methodologies have been developed for culturing intestinal epithelial cells, which are reliant on custom-made components in the cell culture medium. To develop a highly reproducible system with the potential of being widely available to the research field, we took advantage of a commercial medium, Intesticult™ that made it possible to expand and maintain primary human intestinal epithelial organoids. The transfer of organoids from the custom-made medium to the commercially available medium required 2–3 passages before the cultures stabilized, and organoids could subsequently be passaged robustly on a weekly basis (Figure 2a). By RNA-seq, we analyzed changes in gene expression in organoids grown in respective media. We found that 1,934 genes were differentially expressed between cultures maintained in the two different media compositions (absolute log_2_ fold change (log_2_FC) >1; *FDR* < 0.05, by limma analysis). Of these, 1,195 genes had significantly higher expression in the custom-made medium and 739 had higher expression in Intesticult™. Gene ontology (GO) analysis of the genes upregulated in cultures maintained in custom-made medium showed an enrichment of GO terms associated with different metabolic processes, including RNA biosynthesis (Figure 2b, yellow bars and Table S1), while genes upregulated in organoids cultured in Intesticult™ showed a pronounced enrichment of GO terms associated with cell–cell communication and Wnt signaling (Figure 2b, orange bars and Table S1). In line with the enrichment of Wnt signaling components in the organoids cultured in Intesticult™, and the fact that Wnt is an essential signaling component for intestinal stem cell self-renewal both *in vivo* and *in vitro*,^22,23^ these cultures also showed a pronounced enrichment of genes associated with intestinal epithelial stem cells^24^ (Figure 2c;*P* < 2 × 10^–16^, hypergeometric test).

(a) Representative images of organoids cultured according to Sato et al., 2011 (Sato medium) or using Intesticult^TM^ medium with insert showing the morphology of organoids grown under different conditions. (b) The top five Gene Ontology terms enriched in genes upregulated in each media type (indicated by color). X axis shows enrichment by associated-log10 (adjusted *P* value). Rows show ontology terms – for full lists see Table S1. (c) Venn diagram shows the overlap of an established a stem cell signature consisting of 326 genes (white circle,24) with the 994 uniquely annotated genes upregulated in Sato medium (yellow circle) and the 600 uniquely annotated genes upregulated in IntesticultTM medium (orange circle). *P* value is from a hypergeometric test for the larger overlap (56 genes).

### Optimization of 2D culture methods for primary intestinal epithelial cells

The topology of organoids complicates experiments aimed at studying the interactions between the luminal-facing apical membrane of the intestinal epithelial cells and microbes, as the apical side is oriented toward the organoid center, and thus not easily exposed to microbes. We reasoned that growing organoids on a flat surface in 2D would be beneficial since the apical side would be accessible. To assess the behavior of primary epithelial cells in a 2D context, organoids were disaggregated and plated on different extracellular matrix proteins (matrigel, fibronectin, collagen types 1 and 4, laminin 111, 121, 211, 221, 411, 421, 511, and 521). All the tested matrix components supported the establishment of large epithelial sheets (Supplementary Data Figure S1a). Given that laminin 511 and 521 are predominantly associated with the villus basement membrane,^25^ and that the villus is the major site for epithelial microbe interactions, we choose to test Laminin 511 further for its effect on the epithelial cell compartment.

**Figure 3.**
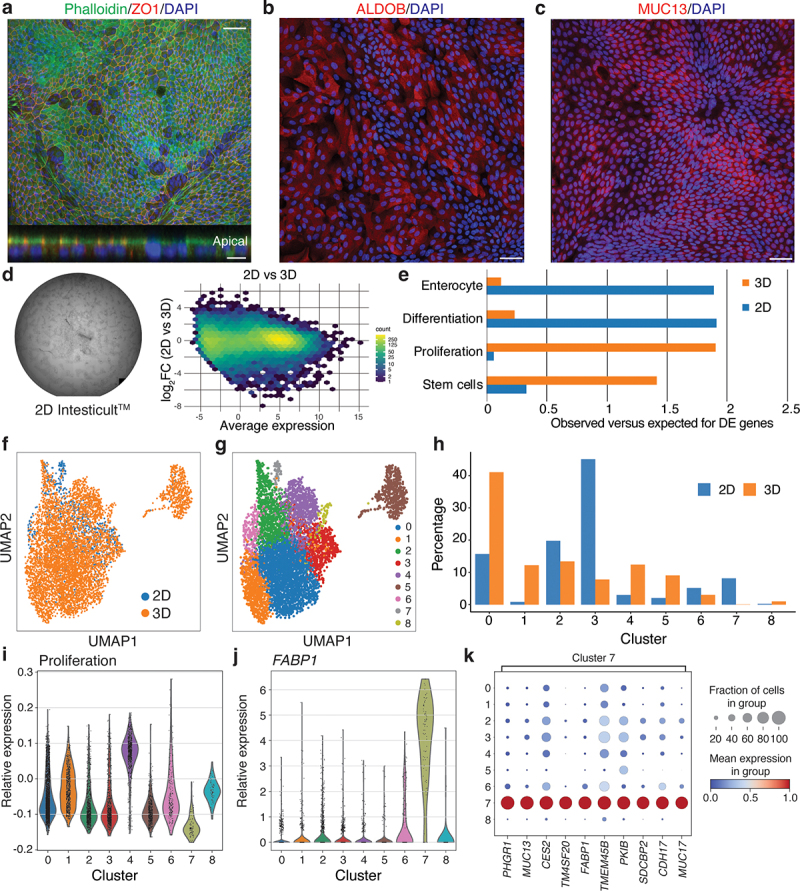
Development and characterization of a 2D model for primary intestinal epithelial cells.

We found that epithelial cells grown on laminin 511 generated a confluent layer with polarized localization of both F-actin and ZO1 to cell surface facing the culture medium, clearly demonstrating the formation of a polarized epithelium with appropriate localization of tight junctions (Figure 3a). The barrier integrity was further supported by trans-epithelial electrical resistance (TEER) measurement (Supplementary Data Figure S1b). In line with the association of laminin 511 with the differentiated compartment in vivo, the 2D epithelium showed large patches of cells expressing markers of the enterocyte (ALDOB) and goblet cell (MUC13) lineages (Figure 3b,c). Next, we compared gene expression profiles of confluent cultures of cell grown in 2D on laminin 511 and in Matrigel as 3D cultures (as described above) and found large differences: 10667 genes were differentially expressed (abs (log_2_FC) >1; *FDR* < 0.05; Figure 3d). Genes upregulated in epithelial cells cultured in 2D were enriched for GO-terms associated with catabolic processes, whereas genes upregulated in 3D cells were enriched for GO-terms associated with general metabolism and cell cycle (Table S2). Aligned with the pattern of protein expression, gene set enrichment analysis revealed that the 2D cultures were enriched for genes associated with differentiation (*P* = 5 × 10^−11^) and underrepresented by genes normally associated with proliferation (*FDR* = 4 × 10^−46^) and stemness (*FDR* = 3 × 10^−20^), while organoids cultured in 3D had the opposite pattern (Figure 3e). Notably, we also observed an enrichment of genes involved in enterocyte differentiation in 2D but not 3D culture (Figure 3e). In line with the expression of markers of terminal differentiation, and the observation that cells cultured in 2D cannot be passaged further strongly suggests that cells within the 2D cultures mimics cell states associated with terminal differentiation.

(a-c) Detection of tight junction marker, ZO1 (red), F-actin with phalloidin (green), and nuclei, DAPI (blue) (a), enterocyte marker, ALDOB (red), and nuclei, DAPI (blue) (b) and goblet cell marker, MUC13 (red), and nuclei, DAPI (blue) (c) in confluent layers of intestinal epithelial cells. Scale bar 25 µm. Insert in (a) shows a Z-section of the cell layer with clear localization of ZO1 and F-actin at the apical surface. Scale bar 10 µm. (d) Left: Primary epithelial cells seeded in 2D form a confluent layer within 7 days from seeding. Right: MA-plot based on RNA-seq analysis of 2D vs 3D cells. Y axis shows 2D vs 3D log_2_FC, and X shows baseline expression in TPM (transcript per million). Color shows the number of genes in each bin. Full list of GO-terms enriched in 2D and 3D cultures in Table S2. (e) Gene set enrichment analysis of the uniquely annotated genes associated with 2D or 3D cultures versus published gene signatures representing enterocyte differentiation, general differentiation in the intestine, proliferation, and stem cells. X axis show observed vs expected overlap based on randomly selected genes. (f,g) UMAP plots of single-cell RNAseq data acquired using the 10× platform. c: Colors show cells cultured in 2D and 3D. d: Colors show nine cell clusters, defined by the Leiden method. (h) Cell distribution between the different clusters from panel j. Bars show a percentage of the entire cell population in the 2D and 3D samples analyzed. (i,j) Relative expression of components of the gene signature analysis for proliferation (i) and the differentiation marker *FABP1* (j) represented as violin plots for each cell population identified by the Leiden clustering. (k) Marker expression analysis identifies a number of differentially expressed genes highly enriched in the differentiation cluster associated primarily with the 2D cell cultures. Colors indicate mean expression; dot size indicates fraction of cells in the group.

Single-cell expression analysis of cells cultured as organoids in 3D or as epithelial sheets in 2D reflected these changes. The combined dataset from cells cultured in 2D and 3D could be divided into nine clusters (Figure 3f–h). Importantly, most clusters were populated by cells from both 2D and 3D cultures, but with different fractions of cells (Figure 3h): clusters 3 and 7 had higher fractions of cells from 2D culture, while clusters 0, 1, 4 and 5 were dominated by cells cultured in 3D. Notably, cluster 1 was almost exclusively populated by cells from 3D cultures, while, conversely, cluster 7 was highly enriched in cells cultured in 2D (Figure 3h). The latter cluster was characterized by high expression of the markers *FABP1*, *MUC17*, and *MUC13* generally associated with differentiation (Figure 3i–k). Cluster 4, which included 2D and 3D cells, was defined by the expression of proliferation markers (Figure 3i).

Thus, by culturing intestinal epithelial cells on laminin 511, it is possible to generate an epithelial cell layer mimicking the differentiated intestinal epithelium exposed to microbes *in vivo*.

### Development of co-culture system for studying interactions between epithelium and microbes

Since we found that epithelial cells cultured in 2D mimic cells in differentiated states, we asked whether it would be possible to exploit this property to assess the interaction between epithelial cells and bacteria, and whether different kinds of bacteria, including probiotic and pathogenic strains, would elicit different types of responses.

**Figure 4.**
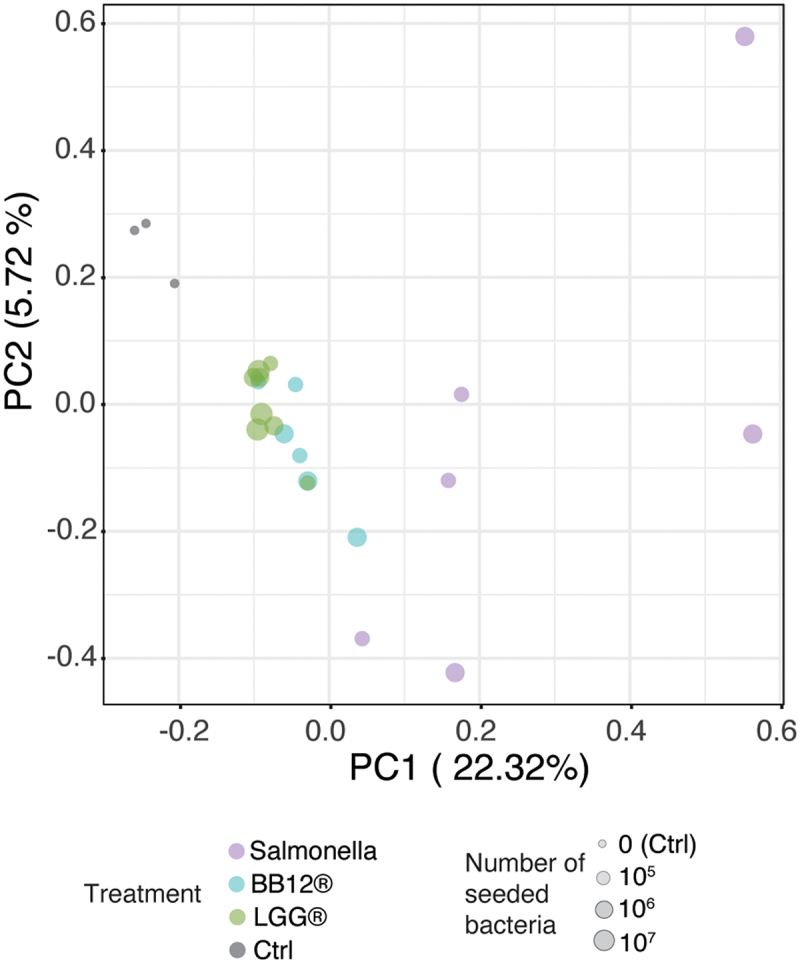
Response to coculture with different microbes.

Therefore, as a first step, organoids from a single individual were dissociated into single cells and cultured in 2D until a confluent monolayer was formed. Importantly, the conductive potential of this confluent layer could be modified upon exposure to the LGG, as evidenced by the TEER assay (Supplementary Data Figure S1b). To assess general responses upon exposure to different types of bacteria, epithelial cells were cocultured with two different probiotic strains (LGG^Ⓡ^, *Bifidobacterium animalis* subsp. *lactis*, BB-12^Ⓡ^ (DSM15954), hereafter called BB-12^Ⓡ^) and *Salmonella typhimurium*, a pathogenic bacterial strain expected to elicit a robust response in epithelial cells. Two hours after the exposure to different titers of either LGG^Ⓡ^, BB-12^Ⓡ^ (both 10^5^, 10^6^, and 10^7^ CFU/well) or Salmonella (both 10^5^ and 10^6^ CFU/well), RNA was isolated for downstream analysis. Co-culturing with bacteria affected the transcriptional state of the epithelial cells, where most of the variance in the dataset originated from the difference between cellular response to salmonella versus non-salmonella strains (Figure 4). Notably, nonpathogenic species (LGG^Ⓡ^ and BB-12^Ⓡ^) formed a gradient between nontreated (ctrl) and Salmonella, and Salmonella species elicited a much wider spread of responses compared to other strains.

Principal component analysis (PCA) of RNAseq data from cultures that stimulated with either LGG^Ⓡ^, BB-12^Ⓡ^, or *Salmonella typhimurium* (indicated by color) across a range of different seeding densities (indicated by point size). X and Y axes show PC1 and 2, variance is indicated at respective PC.

Thus, this initial study demonstrated that it was possible to investigate host responses to microbes using intestinal epithelial cells in this coculture model.

### Epithelial responses to probiotic bacteria

Next, we wanted to extend the initial co-culture study to characterize the acute responses to different probiotic strains of bacteria – LGG^Ⓡ^, BB-12^Ⓡ^, *Bifidobacterium longum* subsp. *infantis*, ISTILOS^TM^ (DSM33361), hereafter referred to as DSM33361, and *Bifidobacterium breve* Bif195, GALENEX^TM^ (DSM33360), hereafter called Bif195 – across cells derived from different individuals.

**Figure 5.**
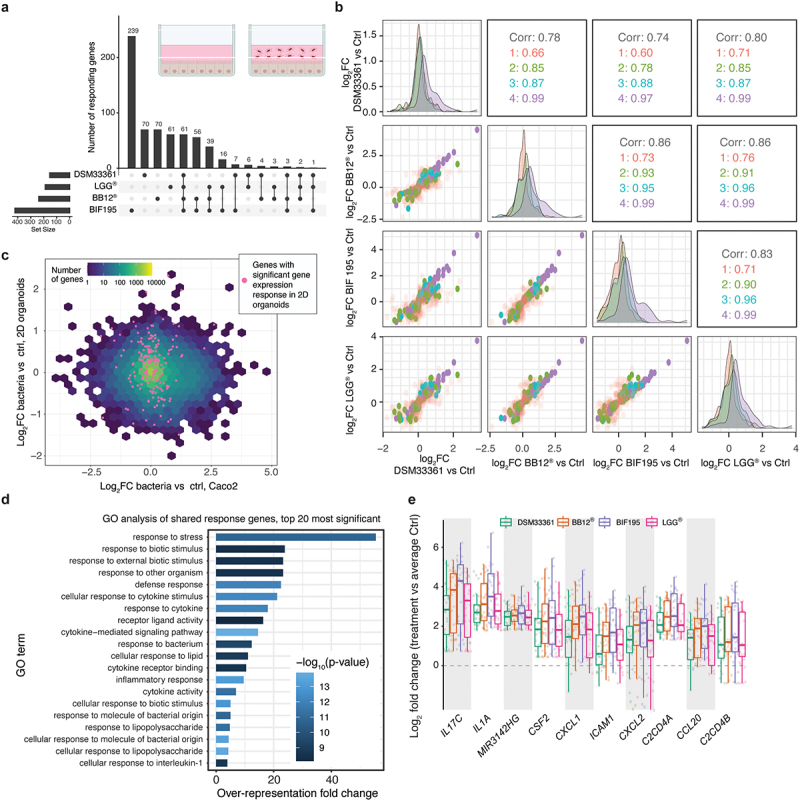
Screening bacteria using 2D model for primary intestinal epithelial cells.

Cells from organoids derived from nine different individuals were seeded on Laminin 511 coated dishes and allowed to grow for 7 days thereby reaching confluence. For each of the four bacteria, confluent cultures were incubated with 10^6^ CFU per well for 2 h at 37°C (Figure 5a, inset). RNA was subsequently isolated to assess the impact of the individual strains on gene expression.

(a) Overlap of differentially expressed genes between bacterial responses from nine individuals, using an UpSet plot visualization. The insert depicts the experimental setup. The upper bar plot shows the number of significantly changing genes (*FDR* < 0.05; log_2_FC > 0.5) in a given set, defined by the lower dot chart. Sets are defined by intersections of the four strain treatments (indicated to the left with total number of differentially expressed genes per set). A single dot indicates genes that are only differentially expressed in one treatment compared to control, while dots connected by lines defined intersects between treatments. (b) Pairwise correlations between gene expression change following bacterial exposure of 2D cultures. X and Y axes show the RNA-seq log2FC response (treatment vs control) of a given bacterial treatment. Dots indicate differentially expressed genes from panel a, colored by whether they are differentially expressed in 1, 2, 3 or 4 treatments. Density plots across the diagonal show the overall distribution of each set and treatment combination. Numbers in remaining cells show the overall Spearman correlation (black) using all points of a given treatment comparison, and Spearman correlations of the subsets of genes defined above. (c) Binned correlation plot comparing the mean log_2_ fold response to all bacterial treatments between Caco-2 cells (x-axis) and primary epithelial cells (y-axis). Hex color shows the number of genes within each hex. Cyan dots show the expression of genes that are differentially expressed in organoids in at least two bacterial strain treatments. Note that these are distributed around 0 log_2_FC in the Caco-2 comparison. (d) Top 20 GO terms enriched for the differentially expressed genes shared between all treatments. X axis shows over-representation fold change. Bar color corresponds to significance (-log_10_ FDR). Full list of GO-terms enriched in Table S3. (e) Expression fold change distribution relative to average control expression for the 10 most differentially expressed genes of the shared set upon stimulation with the four types of bacteria. Y axis shows expression fold change, dots indicate individuals, and color indicates treatment. X axis shows gene names.

We noticed a strong correlation between responses to each bacterial strain across organoid lines derived from all nine individuals. Bif195 drove the strongest response with >400 differentially expressed genes, whereas ~200 genes were differentially expressed upon exposure to BB-12^Ⓡ^, LGG^Ⓡ^ and DSM33361. Both LGG^Ⓡ^ and Bif195 showed strong average responses compared to DSM33361 and BB-12^Ⓡ^. Pairwise contrasts, comparing bacteria treated and non-treated primary epithelial cells from the same individual, showed that more than 50% of differentially expressed genes (*FDR* < 0.05 and |log2FC|) > 0.5) were affected by at least 2 strains, including a common shared response of 61 genes that were differentially expressed following exposure to all four probiotic strains (Figure 5a). To assess the similarity in responses between bacteria more quantitatively, we calculated the control vs bacterial exposure log_2_FC for each gene according to treatment, and then compared the bacterial response by performing Spearman correlations between pairs of bacteria treatments using these log_2_FC values. In general, all pairwise comparisons showed overall strong correlations (0.7–0.86) (Figure 5b). To extend this, we remade the analysis by only focusing on genes that were differentially expressed in one (Figure 5b, red label), two (green label), three (cyan label), or all four strains (purple label). As expected, genes with strain-specific responses had the lowest correlation across strains (~0.7), while genes shared between all strains had the highest (~0.99). However, genes with a significant response in at least 2 strains retained a very high correlation (~0.9) (Figure 5b), even in strains that did not show significant responses for those genes. These findings suggested that genes involved in responses in two strains are most likely affected across all treatments, and that the observed differences in their response between strains were most likely caused by the magnitude of the differential expression, or variance across individuals, rather than by a true lack of response. Consistently with this hypothesis, we observed that strains, which induced higher average responses (Bif195 and LGG^Ⓡ^) showed higher numbers of significantly differentially expressed genes.

Confluent layers of Caco-2 cells have been the traditional assay for interrogating interactions between intestinal epithelial cells and bacteria by stimulation for, e.g., 24 or 48 h.^26^ To compare the response between primary intestinal epithelial cells and the Caco-2 cells, we performed parallel experiments with the four different bacterial strains using confluent layers of Caco-2 cells exposed to the same bacteria for 2 h. In contrast to the analysis of primary epithelial cells, we detected only a handful of differentially expressed genes (LGG^Ⓡ^: four genes; DSM33361: five genes; BB-12^Ⓡ^: four genes; Bif195: two genes; Bif195 and BB-12^Ⓡ^: two genes), with lower correlations in the response to different strains when compared to the 2D organoids (Supplementary Data Figure S2a). Notably, there was no overlap between the differentially expressed genes identified in the analysis of Caco-2 cells and primary epithelial cells and no correlation between their fold change (Figure 5c), even though baseline expression of all genes was highly similar between Caco-2 cells and organoids (Supplementary Data Figure S2b). Thus, in contrast to Caco-2, which exhibited a minimal acute response to bacteria, primary epithelial cell cultures growing in 2D represented a robust method for assessing epithelial–bacterial interactions.

To characterize the shared response in intestinal epithelial cells in greater detail, we performed GO analysis on the gene signature of 61 genes that were differentially expressed following all bacteria treatments (shared response). As expected, this signature was highly enriched for genes associated with cytokine signaling, response to lipopolysaccharides and molecules of bacterial origin (Figure 5d, Table S3). The 18 most differentially expressed genes across the 4 bacterial strains and across the 9 individuals, contained 9 secreted inflammatory signaling molecules (*IL-17C, IL-1A, CSF2, CXCL1, CXCL2, CCL20, CXCL3, TNF, CXCL8*) implicated in bacterial sensing.^27^ Importantly, these displayed responses of between two- and fourfold expression increases compared to control treatment (Figure 5e). This suggests that these probiotic strains do not induce acute inflammation per se but rather an increased state of immune surveillance. In contrast, none of the genes differentially expressed only in response to one strain showed significant GO term enrichment.

Collectively, this illustrates that the established 2D system for primary epithelial cells is more sensitive to microbe co-incubation compared to Caco-2 cells, and thus primary epithelial cells enable more nuanced studies of the acute response to microbes.

## Discussion

Here, we present a versatile method for culturing intestinal epithelial cells as confluent monolayers that enable assaying bacteria for their interactions with the epithelium. We demonstrate that epithelial cells grown as a confluent monolayer on laminin 511 lose their proliferative profile and stem cell features, and up-regulate genes associated with differentiation and in particular along the enterocyte lineage. This is a robust response observed across multiple primary organoid lines treated in the same manner and provides a model system that enables *in vitro* studies with primary human epithelial cells aimed at mapping interactions with microbes and potentially microbe-derived metabolites and surface markers. The methodology enables both scale-out and scale-up to assay larger effects in pathogenic strains of bacteria or smaller, yet critical functions of how epithelial cells respond to bacteria used in food supplements, as well as trafficking across the epithelial lining of the intestine. Importantly, here we observe conserved effects upon exposure to different strains of microbes across individuals and including a shared set of genes. Importantly, while primary cells cultured in 2D elicited a robust response, the prevailing model for probiotic-host cell interaction, the Caco-2 cell line, showed little, if any, acute response to the same microbial strains.

Traditionally, microbial responses have been studied in cancer cell lines and strains exhibiting a given response have been tested using various mouse models.^28^ This has provided physiological models for developing insights into the processes that shape the development of microbiome diversity and host responses.^29^ Although essential knowledge related to the physiology of host–microbiome interactions can be obtained from studies using mouse models, there are significant differences when compared to humans that cannot be encompassed in such *in vivo* models. Studies of specific microbial responses in intestinal epithelial cells have consequently searched for new alternatives of human origin. Here, intestinal epithelial organoids derived from single induced pluripotent stem cell lines have proven to be valid *in vitro* models, although the 3D nature of cells cultured as organoids has proven to complicate the development of high-throughput methodology.^11,30^ Moreover, it is also worth pointing out that in addition to the obvious variation in the microbiome between individuals, the response to microbes and the susceptibility to aberrant effects upon exposure to specific strains of microbes are influenced by the genetics of the host.^31^ To address potential individualized responses, it is essential to establish tractable model systems that can encapsulate such differences. The 2D culture system presented here demonstrates that primary epithelial cells isolated from human intestinal biopsies and cultured in 2D represent a robust methodology to assess microbial responses across a population.

Natural genetic variance has formed the basis for precision medicine for human disease using, e.g., patient-derived organoids for drug screening.^32,33^ It is evident that an appropriate composition of microbes provides important functions supporting digestion and immune imprinting.^34^ It does, however, remain unclear whether the genetic variance provides specific microbial responses, which can now be determined with the established method. Although some microbes tolerate an aerobic environment, the majority of intestinal microbes are obligate anaerobic. Further methods development should consequently include the introduction of systems devoid of oxygen, in which short term is likely to be complementary to the cultures of differentiated epithelial cells.

## Materials and methods

### Human subjects and ethics

The cohort used for the derivation of organoids has been described in a previous study.^19^ It includes healthy individuals (males and females) between 18 and 35 years old with a BMI <30 kg/m^2^. Participants provided written consent following both oral and written information about experimental procedures. The Danish regional ethics committee approved the study (H-17002470), which has been performed in accordance with the Declaration of Helsinki. No severe adverse events were observed during the study. All data analyses were performed blinded. The trial was registered at ClincialTrials.gov with the identifier NCT03140878. Collection and cultures of biopsies from the human colon were approved by the ”Danish Nationa Research Ethics Committee” (CVK-1302159).

### Organoid growth

Epithelial organoids were established from small biopsies obtained from the duodenum and jejunum from healthy individuals as described.^9,19^ Briefly, two biopsies were washed briefly in PBS prior to incubation in 2 mM EDTA for 30 min at 4°C. Crypts were subsequently isolated by repeated pipetting, isolated crypts were seeded in Matrigel (Corning) and cultured in advanced DMEM/F12 supplemented with Glutamax, Pen/strep, HEPES, B27, N2, Noggin, EGF, human recombinant R-spondin 1 (500 µg/mL), N-acetyl-L-cysteine, Nicotinamide, A83-01, SB202190, PGE2, and 50% conditioned WNT3A medium. Moreover, for the first 3 days following seeding, the medium was supplemented with Y-27632 (10 µM). Cultures were passaged every 7–10 days by dissociating the matrigel in cell recovery solution (StemCellTech).

The transfer from the custom-made medium into Intesticult medium (StemCellTech) required 2–3 passages of adaptation. The cells were subsequently cultured in accordance with manufacturer’s instructions.

### Establishing 2D cultures for intestinal epithelial cells

Fragments from organoids were transferred into wells coated with different extracellular matrices diluted in PBS. This included Matrigel (Corning; 1:100 dilution), Collagen type I (Corning; 1:100), Collagen IV (Sigma; 10 µg/cm2), Fibronectin (Sigma; 5 µg/cm2), and eight different kinds of laminins; Laminin 521, Laminin 511, Laminin 421, Laminin 411, Laminin 221, Laminin 211, Laminin 121, and Laminin 111 all coated at 2.79 µg/cm^2^. The epithelial fragments were seeded in complete custom-made medium as described above and cultured in a 37°C CO_2_ incubator for 7 days with a complete media change on days 3 and 5. The plates was subsequently imaged using an EVOS® FL Auto Imaging System (Thermo Fisher Scientific).

### Preparation of bacteria for coculture experiments

Bacterial solutions were freshly prepared for all experiments. Freeze dried preparations of LGG^Ⓡ^, DSM33361, BB-12^Ⓡ^, Bif195, and Salmonella (approximately 0.1 g) were dissolved in 10 ml PBS supplemented with 0.05% L-Cysteine hydrochloride (CyHCl). To remove cryopreservants, the solution was diluted 1:10 in 0.05% CyHCl PBS and bacteria were pelleted at 2500 g for 4 min. The pelleted bacteria were resuspended in 10 mL 0.05% CyHCl PBS and the centrifugation was repeated. This was repeated with PBS before resuspending the bacterial pellet in 200 µL of Intesticult 2D media supplemented with Y27632. To normalize the CFU counts, individual bacterial solutions were adjusted to OD_600_ = 1 (LGG^Ⓡ^ OD_600_ = 1.0 (1.2 × 10^5^ CFU/µl); DSM33361 OD_600_ = 1.0 (2.6 × 10^5^ CFU/µl); BB-12^Ⓡ^ OD_600_ = 1.0 (1.4 × 10^5^ CFU/µl); Bif195 OD_600_ = 1.0 (2.2 × 10^5^ CFU/µl)).

### 2D co-culture with bacteria

For coculture experiments, the appropriate number of individual wells in Primaria 96-well plates (Corning) were coated with 0.79 µg/cm^2^ Laminin 511 (Biolamina) resuspended in PBS overnight at 4°C. Organoids were grown for 7 days in Intesticult (A+B) supplemented with Penicillin–Streptomycin (1%) in domes of matrigel in 48 well plates. Epithelial cells from one droplet of Matrigel were sufficient for culturing one well in a 96 well plate in 2D corresponding to approximately 250,000 single viable cells. Briefly, droplets were collected in gentle cell dissociation reagent (Stem Cell Technologies) and allowed to incubate for 12 min at RT on a rocking table to release organoids. Organoids were allowed to sediment at the bottom of the tube, the supernatant was removed and new DMEM/F12 was added, before the tube was spun at 200 g for 5 min. After removing the supernatant, 0.05% prewarmed TrypLe (Gibco) was added, and the suspension was incubated at 37°C for 5 min followed by vigorous pipetting to generate a single-cell suspension. Fresh DMEM/F12 was added, and the tube was spun at 200 g for 5 min. Pelleted epithelial cells were resuspended in 100 µL of Intesticult (Stem Cell Technologies) supplemented with Penicillin–Streptomycin (1%) and Y27632 (10 µM) and seeded in the Laminin 511 coated wells. To ensure that all epithelial cells were sedimenting at the bottom of the wells, plates were spun down at 200 g for 3 min. The plates were placed in a CO_2_ incubator at 37°C for 24 h before changing medium to Intesticult (Stem Cell Technologies) supplemented with only Penicillin–Streptomycin (1%). Media was subsequently changed on day 3 and day 5, and the wells were confluent on day 7 and ready for coculturing.

For the cocultures, the medium was removed and each well washed briefly with PBS at room temperature. The PBS was removed and 100 µL Intesticult medium without Penicillin-Streptomycin was added to control wells, whereas Intesticult medium containing bacteria was added to wells designated for coculturing. The plates were spun briefly at 300 g for 3 min and incubated at 37°C for 2 h in the CO_2_ incubator. After 2 h, the wells were washed and epithelial cells lysed for RNA purification.

### TEER measurements on human SI organoids treated with LGG®

Intestinal epithelial organoids were harvested and disaggregated into a single-cell suspension by incubating with TrypLe. The single-cell suspension was subsequently transferred to clear 24 well transwell inserts with a 0.4 µm polyester membrane (Corning) coated with 0.79 µg/cm^2^ laminin 511 (Biolamina) for 48 h at 4°C. Each 24 well transwell was seeded with organoids from a 25 µl matrigel dome corresponding to 250,000 viable single cells. Following 1 week of culturing, a medium without Penicillin-Streptomycin was added (950 µl basolateral and 190 µl apical) to the wells and inserts were transferred to the CellZscope2. TEER measurements were subsequently performed every hour for 16 h to establish baseline TEER. 10^6^ LGG^Ⓡ^ was added per well for continued measurements for an additional 24 h.

### Caco-2 cell maintenance and differentiation

Caco-2 cells were maintained in DMEM (1×) Glutamine (21885-025) with 1% Non-Essential Amino Acids Solution (SH30238.01), 20% Fetal Bovine Serum (heat-treated) (10500-064), and 1% Pen-Strep solution (03–033-1B). The cells were passaged once reaching 50% confluency. Prior to co-culture experiments 2 × 10^4^ cells/well were seeded in 15 wells of a 96-well plate. Cells were 100% confluent on day 3, and co-culture with all four probiotic strains was performed on day 7 in the same manner as for 2D primary cells described in this paper.

### Antibody staining

For antibody staining, confluent cell layers were preincubated in BSA (5%) supplemented with TritonX100 (0.1%). Primary antibodies were incubated O/N at 4°C in blocking buffer. The following primary antibodies were used: anti-ZO1 (61-7300; Invitrogen), anti Muc13 (HPA045163; Atlas Antibodies), and anti-AldoB (HPA073201; Atlas Antibodies) and secondary antibodies Alexa Fluor 647 polyclonal donkey anti-rabbit. The material was counterstained with Alexa Fluor 568 conjugated-Phalloidin and/or DAPI.

### RNAseq analyses

RNA extraction and sequencing was performed on two batches of samples, one for cells grown in noncommercial media and one for cells grown in Intesticult™ media. The analysis included sequencing of 354 Illumina paired-end libraries for cells cultured in Intesticult™ media (read length 150 bp), with an average 47 million high-quality reads (min 40, max 70), and 188 unpaired Illumina libraries for cell lines cultured in noncommercial media with an average of 6.4 million high-quality reads (min 4.9, max 8.6). The quality of reads across all libraries was assessed using fastqc and multiqc. To remove adapter bias, the first 11 bps were trimmed from reads from the noncommercial media libraries and the first 15 bp from reads from the Intesticult™ libraries using the trimfq function of seqtk. Base-pair bias was also detected in the last 2 bps of Intesticult™ reads, and these 2bps were removed accordingly.

All reads were mapped to the version 32 of the Human Genome Annotation (GRCh38) using Salmon version 1.1.0, and transcriptome index was built with k-mer length of 31, using the decoy-aware option to account for potential spurious matches from unannotated genomic regions. Pseudomapping was performed using selective alignment in Salmon quant with 10 bootstraps, limiting the minimum score fraction for matching reads to 0.8, correcting for GC and sequence-specific bias. Transcript information was annotated using tixmeta, and TMM normalization was performed followed by Voom transformation for analysis with the limma package. Due to the large differences in variance between Caco-2 cells and organoids, normalization and analyses were performed separately for each of the two cell types. Only genes with more than five counts in at least 70% of the samples in one condition of our experimental design were included in the differential expression analysis. In both organoids and Caco-2 cells, the final linear model included a single-factor design that compared the effect of each bacterial treatment to the control samples. To account for correlation in expression between organoid lines sampled from the same individuals, genotype was included as a blocking factor in both Voom normalization and linear model fitting.

Finally, multiple-hypothesis testing was corrected using stage-wise analysis as implemented in the R package stageR.^35^ Briefly, stage-wise analysis is divided into two stages. The first selects all genes that pass a significance threshold for a given factor (in our case, *p* < 0.05 for bacterial treatment). The second applies p-value adjustment to the set of remaining factors, in order to detect which specific levels (in our case bacterial strains) are responsible for its significance.

### Single cell library preparation and sequencing

Single-cell libraries were prepared using the 10X Genomics protocols v2 Chemistry. A maximum of 20,000 sorting events from a BD FACSAria sorter were loaded per well in a volume of 33.8 μL ultra clean 0.1% BSA/PBS. Cells were encapsulated in droplets of Gel Bead-in-Emulsions (GEMs) using the 10X Genomic Single Cell Chip. Reverse transcriptase was performed after the GEMs formation. cDNA was purified and amplified with 12 PCR cycles. Libraries were diluted to 2 nM in elution buffer, and two libraries were pooled and run on the same flow cell. The libraries were sequenced on an Illumina NextSeq 500 platform with a High Output 150 cycles kit.

### scRNAseq analysis

Cell Ranger (v3.0.1) software from 10× Genomics was used to process Chromium single-cell RNA-seq output to align reads and generate bam files.^36^ Reads were mapped to the refdata-cellranger-GRCh38–3.0.0 reference downloaded from the 10× Genomics website (https://support.10xgenomics.com/). One of the Cell Ranger outputs were bam files, which were then processed with velocyto using the run10× function.^37^ Velocyto counted reads falling into exonic/intronic regions and generated spliced/unspliced expression matrices in a loom file.

The loom files were processed and analyzed in Python using scanpy^38^ and scVelo.^39^ First, low-quality cells were filtered out based on the distributions of spliced counts, unspliced counts, and genes. Next, cells with a high ratio of counts originating from mitochondrial features (more than 0.4) or potential doublets (two cells in a single bead) were eliminated, and doublets predicted using both scrublet and Doublet Detection.^40,41^

The data from 2D and 3D samples were combined and genes expressed in less than 5 cells or with less than 20 counts were filtered out, followed by normalization and log transformation of the data. For batch correction, the top 2000 highly variable genes were included using the mutual nearest neighbor’s algorithm implemented as mnn_correct function in scanpy’s API. Parameter k was set to 15 and var_adj to True. The proliferation score was annotated before the batch correction using the score_genes_cell_cycle function from scanpy, based on a published signature.^42^ Variation arising from the cell cycle stage was reduced by linearly regressing the annotated S and G2/M scores.^43^ Afterward, the dataset was dimensionally reduced first with principal-component analysis (PCA) and then with Uniform Manifold Approximation and Projection (UMAP). Cell neighbors were calculated using a batch balanced k-nearest neighbors algorithm with default parameters.^44^ Finally, the cells were clustered in an unsupervised manner using the Leiden algorithm.^45^ Then, differentially expressed genes (DEGs) in the clusters were detected with the Wilcoxon rank sum test using scanpy’s rank_genes_groups function.

## Supplementary Material

Supplemental Material

## Data Availability

The expression data from CaCO2 cells stimulated with bacterial isolates are deposited at the NCBI Gene Expression Omnibus (GEO) under accession number GSE231605. https://www.ncbi.nlm.nih.gov/geo/query/acc.cgi?acc=GSE231605
