# Supplementary figures and images for: Detecting host responses to microbial stimulation using primary epithelial organoids
Source: Gut Microbes. 2023 Nov 22;15(2):2281012. doi: 10.1080/19490976.2023.2281012 (PMC10730191; doi:10.1080/19490976.2023.2281012)

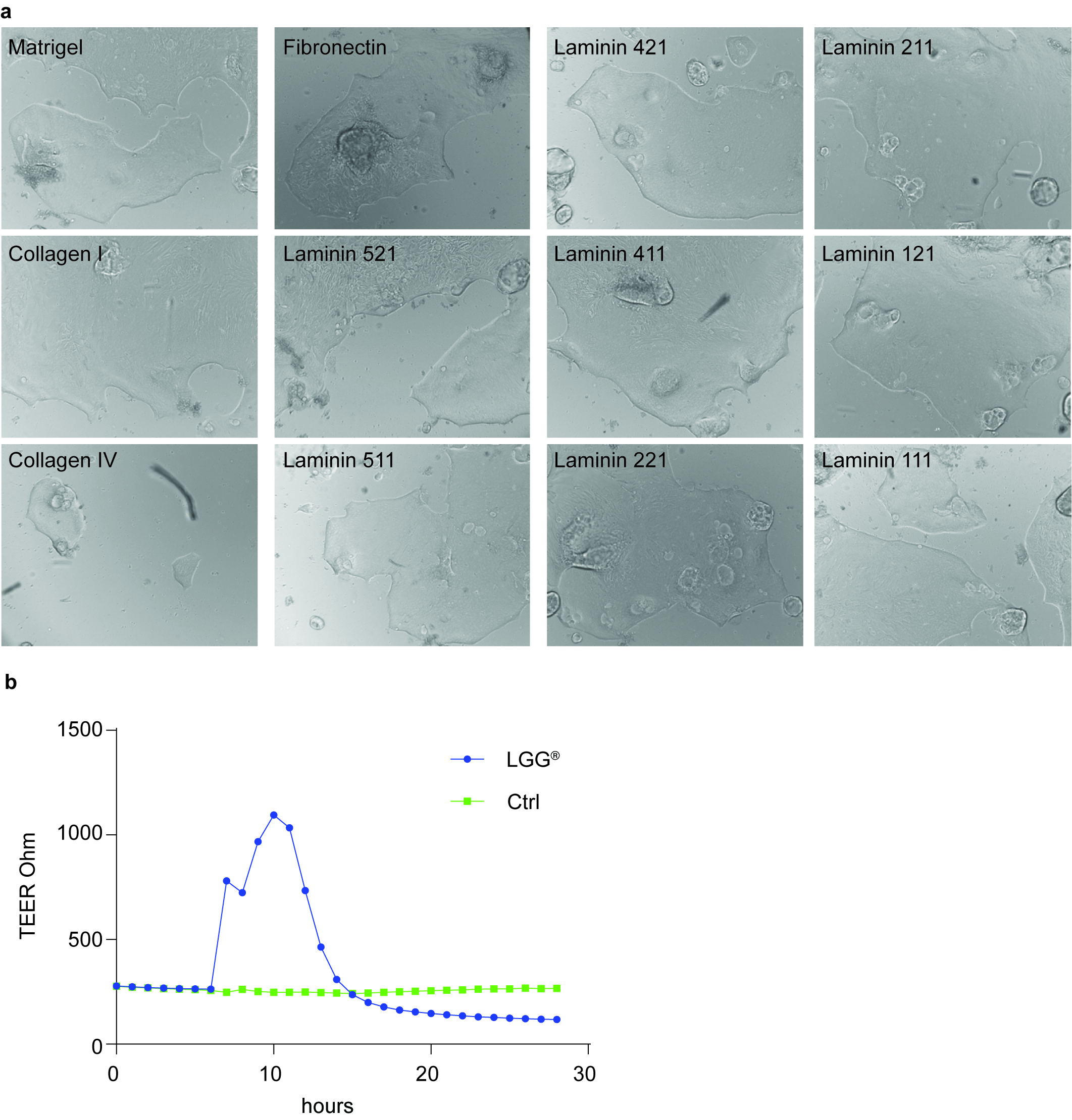

Supplement: Supplemental Material [file KGMI_A_2281012_SM9287.zip › FigureS1.tif]

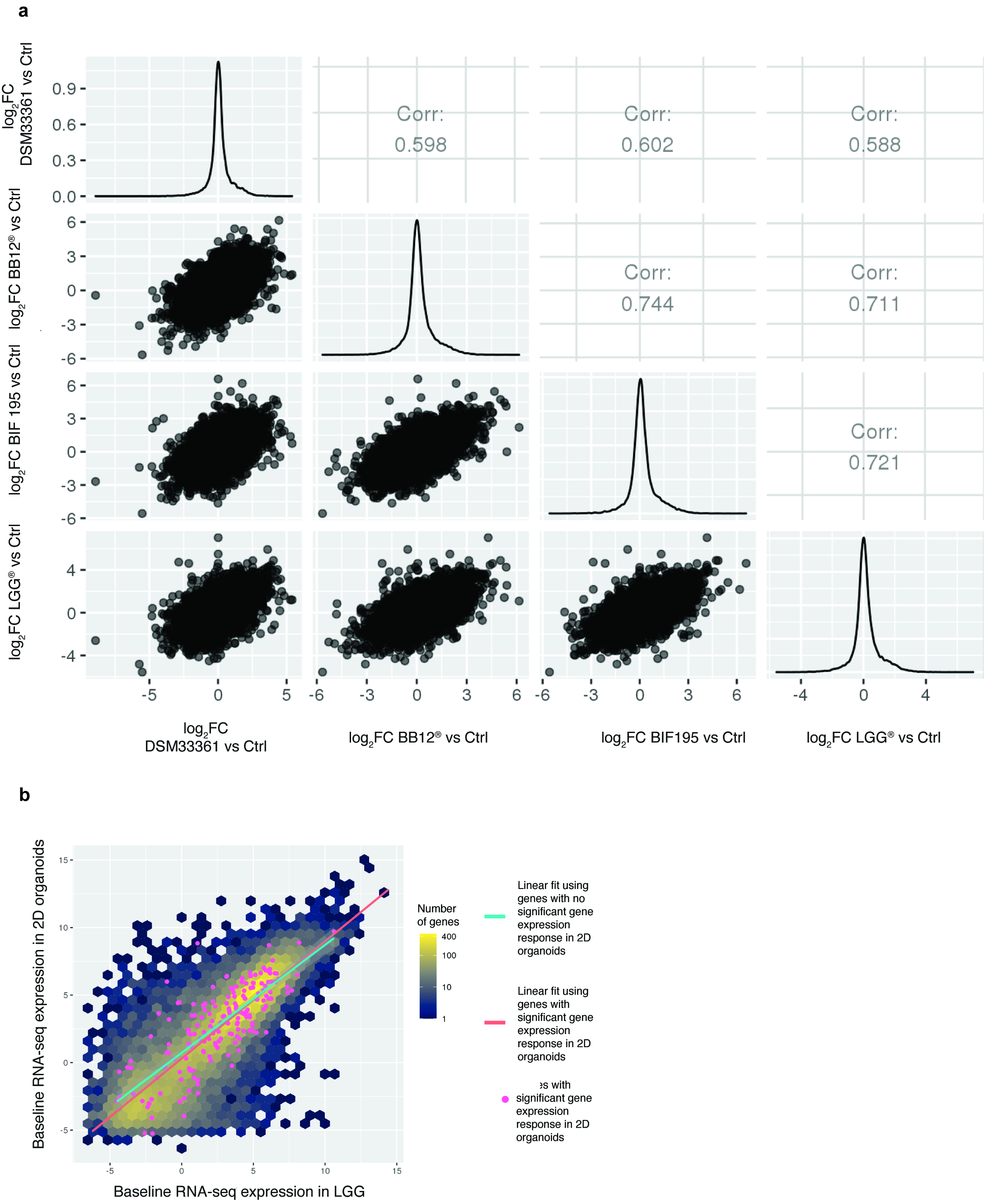

Supplement: Supplemental Material [file KGMI_A_2281012_SM9287.zip › FigureS2.tif]
